# Editorial Correspondence

**Published:** 1857-01

**Authors:** W. F. Westmoreland

**Affiliations:** Paris


### EDITORIAL CORRESPONDENCE.

Paris, October 24th, 1856.

*Dear Doctor*—In my letter of the 15th inst., I promised, in my next, to give you, at least, the position of those who had taken part in the discussion now pending at the *Académie de Médecine*, upon the propriety of the injection of iodine in ovarian cysts. The discussion, as I stated in my last, grew out of the report of a very interesting case, by M. Barth, the peculiarities of which I have already given you.

M. Malgaigne was the first to obtain the floor, and gave his views at some length. Said he could see no advantage whatever in M. Barth's plan of operating—that the second puncture was of no use, and in surgery, as a general rule, what was of no use might result in injury. After discussing the usual plans of treatment, he expressed the opinion, that we should not attempt the radical cure—that the injection of iodine or other irritants into ovarian cysts is attended with very great danger. He was content, he said, to leave such cysts to themselves,—making a simple puncture when the symptoms become urgent.

As to the extirpation of ovarian tumors, he considered the idea absurd; had no confidence in the statistics of this operation as presented by American surgeons—regards them, as he does, the statistics of some surgeons in France, who present all their successful cases with great noise, and entirely forget their unsuccessful ones.

M. Moreau said he had witnessed several cases of pregnancy during the existence of an ovarian cyst, which passed through the term without any very unpleasant symptoms; that pregnancy, during an ovarian dropsy, was not so rare as many were disposed to believe. He found the plan of M. Barth's operation very rational; but, like M. Malgaigne, he was not disposed to attempt the radical cure—was content to make a simple puncture for the discharge of the liquid when the symptoms become urgent. In one case, he had seen a radical cure from a simple puncture.

M. Cazeau said he had seen a number of cases treated by the injection of iodine, and in quite the majority, with very happy results. That in cases where the injections of iodine are admissible, there is not the danger that M. Malgaigne would have us believe. That the injections of iodine are only admissible in simple ovarian cysts; that in the multilocular cysts of this organ, the injections of iodine, although sometimes successful, is attended with very great danger.

M. Velpeau considers the subject under discussion of the very greatest importance; that although much has been said upon the treatment of ovarian cysts by the injection of iodine, it had never been seriously discussed. He spoke of the epidemic which had prevailed in America for the extirpation of ovarian tumors, but which, he said, in France, they had had the good sense never to attempt. Was of the opinion that the injection of iodine merited consideration, and should be studied with care. He entered into a discussion of the pathology of ovarian tumors, and their usual course when left to themselves; said that they might exist a number of years without any very unpleasant symptoms; mentioned cases that he had known to exist for thirty and forty years without treatment; was favorably impressed with M. Barth's plan of operating. He expressed the opinion that in simple cysts of the ovary, uncomplicated with tumor, &c., the patient not too much exhausted, the injection of iodine might be attended with success. He has seen one case cured by the injection of iodine when the cyst was multilocular. At the close of his remarks, he moved a continuation of the discussion.

At the next meeting the discussion was continued by M. M. Trousseau and Jobert. M. Trousseau was opposed to the injection of iodine; had never injected an ovarian cyst; considered it extremely dangerous; was content with a simple puncture, which he contended, if made early, before the cyst had lost all its contractility, was not unfrequently followed by a radical cure.

M. Jobert was a warm supporter of the injections of iodine. He said that in his hands it had been followed by the most brilliant results. His plan of operating is to leave a canula in the cyst until there are adhesions between the cyst and the walls of the abdomen, thus preventing the escape of iodine or other fluids into the cavity of the abdomen. Since he has adopted this plan, he has operated on thirty patients without any grave symptoms, and most frequently with permanent cure.

The discussion was continued, and from the interest manifested, as well by members as those in the lobby, will likely be protracted for several weeks. So far, one fact is to be remarked, that no one of those who have opposed the usual plans of treatmeut, proposed for the radical cure of ovarian cysts, have ever attempted the injection of iodine in such cysts.

I visited to-day *La Pitié*, and was pleased to find that M. Maisonneuve had given up his poorly ventilated and contracted service at *Hôpital Cochin* for extensive wards in the above mentioned hospital. As usual, I found his wards crowded with Americans; attracted, perhaps, by the peculiarity and boldness of his operations. M. Maissonneuve is certainly one of the boldest, if not one of the best, operators in Paris, but still does not occupy that position that many believe due him.

I saw him ligate this morning the external carotid artery, for a malignant growth, at the base of the tongue, with the object of staying the progress of the disease, by cutting off the nutrition of the part. He says that in quite a number of cases, where it was not practicable to attack the disease, either with the knife or the cautery, he has ligated the arteries, supplying the parts with very good results. It is in another point of view, however, that I would more particularly call attention to this operation, and that is in the selection of the external carotid, for the ligature, instead of the primitive, which is usually ligated—in fact, which is the only recognised operation by standard works on Surgery, either in Europe or America. One of the principal dangers in ligating the primitive carotid artery, as is well understood, is the disturbance of the functions of the brain, which has, in a number of cases upon record, proved fatal in a very short time, and in others has resulted in hemiplegia of the opposite side, indicating a lesion of that side of the brain, corresponding with the artery ligated. To prevent the possibility of this unpleasant complication, M. Maisonneuve, proposes to ligate the external carotid, instead, of the primitive, in all lesions, where it is desirable to arrest the circulation, in the external carotid. Although he proposed and performed this operation several years ago, (I recollect to have seen him perform it twice in 1853,) it has not received that consideration from the profession, to which I think it justly entitled. He stated this morning, that he had ligated the external carotid fourteen or fifteen times, without the loss of a patient from the operation.

The point selected for the ligature, is just above the bifurcation—between the origin of the superior thyroid and the lingual arteries. After ligating the external carotid, if it be slightly raised, a ligature may be readily passed beneath the superior thyroid, the ligation of which, is necessary, from the proximity of its origin to the point selected for the ligation of the external carotid.

Within the past few days, I have noticed several cases of varicose veins treated with the injection of the per chloride of iron, in the diseased vein. M. M. Broca, and Maisonneuve, in whose wards I have observed the above cases, contend, that with the necessary precautions, there is nothing to be feared from such injections, and that they are attended with very satisfactory results. In the cases that I have noticed, and I suppose I have seen six or eight, there has been no unpleasant symptom—the blood readily coagulating at the point where the injection was made, forming a hard tumor, the size depending upon the quantity of blood contained in the vein, arresting the circulation, and these obliterating the vessel. The precaution important is, to have the vein distended with blood, and to compress the vessel above and below the point selected for the puncture, during, and for several days after the operation. The injection is made with a small graduated syringe, with a metalic point, which is thrust into the vein. From five to fifteen drops is injected, depending upon the quantity of blood to be coagulated. The per chloride of iron, as you are apprised, was in 1853 proposed, upon the same principle, for the treatment of aneurisms, and in a number of cases was injected into aneurismal sacks, with, however, very unfavorable results, several cases proving fatal. It is at present entirely abandoned.

Experiments with carbonic acid still continue; it is rather gaining in importance than otherwise; numerous cases are reported of its happy effect in allaying pain.

The regular clinical Professors will take their places in a few days, when I hope to be able to give you something more interesting. Yours, &c.

W. F. WESTMORELAND.

